# Influence of Residual Disease on the Efficacy of PARP Inhibitors in Advanced Epithelial Ovarian Cancer: A Systematic Review and Meta Analysis

**DOI:** 10.3390/cancers17203365

**Published:** 2025-10-18

**Authors:** Sekyoung Hwang, Ji Hyun Kim, Uisuk Kim, Hyeong In Ha, Sang-Yoon Park, Myong Cheol Lim

**Affiliations:** 1 School of Pharmacy, University College London, London WC1N 1AX, UK; sekyoung.hwang.22@ucl.ac.uk; 2 Center for Gynecologic Cancer, National Cancer Center, Goyang 10408, Republic of Korea; jihyunkim@ncc.re.kr (J.H.K.); uisookkim@ncc.re.kr (U.K.); parksang@ncc.re.kr (S.-Y.P.); 3 Ilsan Hospital, Goyang 10444, Republic of Korea; hi126908111@gmail.com; 4 Rare and Pediatric Cancer Branch and Immuno-Oncology Branch, Division of Rare and Refractory Cancer, Research Institute, National Cancer Center, Goyang 10408, Republic of Korea; 5 Department of Public Health & AI, National Cancer Center Graduate School of Cancer Science and Policy, National Cancer Center, Goyang 10408, Republic of Korea; 6 Department of Cancer Control and Policy, National Cancer Center Graduate School of Cancer Science and Policy, National Cancer Center, Goyang 10408, Republic of Korea

**Keywords:** epithelial ovarian cancer, PARP inhibitors, residual disease, progression-free survival

## Abstract

**Simple Summary:**

This systematic review and meta analysis evaluated six randomized controlled trials including 3629 patients to determine whether residual disease modifies the clinical benefit of PARP inhibitor maintenance therapy in advanced epithelial ovarian cancer. The analysis demonstrated that PARP inhibitors significantly improve progression-free survival in both patients with no gross residual disease (R0; hazard ratio 0.55, 95% confidence interval 0.44–0.68) and those with macroscopic residual disease (R1/R2; hazard ratio 0.51, 95% confidence interval 0.40–0.65). The treatment effect was similar between subgroups (*p* = 0.66). These findings indicate that PARP inhibitor maintenance therapy provides substantial benefit regardless of surgical outcome, supporting its use in all eligible patients after first-line platinum-based chemotherapy, while emphasizing that achieving complete cytoreduction remains vital for the best overall prognosis.

**Abstract:**

Objective: While PARP inhibitors (PARPi) improve progression-free survival (PFS) in advanced ovarian cancer, their efficacy across different surgical outcomes is unclear. We aimed to determine if the efficacy of PARPi maintenance therapy, as measured by PFS, is modified by postoperative residual disease (R0 vs. R1/R2) in newly diagnosed advanced epithelial ovarian cancer. Methods: A systematic review and trial-level meta analysis of randomized controlled trials published through July 2025 was conducted. The primary endpoint was pooled hazard ratio (HR) for PFS, with subgroup analyses based on residual disease (R0 vs. R1/R2), clinical risk (higher risk vs. lower risk), and timing of surgery (primary cytoreductive surgery vs. interval cytoreductive surgery). Results: Six randomized controlled trials involving 3629 patients were included in this meta analysis. PARPi maintenance significantly improved PFS in both patients with no gross residual disease (R0) (HR 0.55, 95% CI 0.44–0.68, I^2^ = 64.2%) and those with macroscopic residual disease (R1/R2) (HR 0.51, 95% CI 0.40–0.65, I^2^ = 56.0%). The treatment effect did not differ significantly between these subgroups (*p* = 0.66). A numerically greater benefit was observed in lower-risk populations (HR 0.40, 95% CI 0.29–0.55, I^2^ = 0.9%) compared to higher-risk populations (HR 0.51, 95% CI 0.36–0.73, I^2^ = 78.5%, *p* = 0.30). The benefit was maintained irrespective of surgical timing, with similar pooled HRs for patients undergoing primary (HR 0.56, 95% CI 0.42–0.74, I^2^ = 72.3%) versus interval (HR 0.54, 95% CI 0.45–0.66, I^2^ = 44.2%) cytoreductive surgery. Conclusions: PARP inhibitor maintenance therapy provides a significant PFS benefit regardless of residual disease status, supporting its use in all eligible patients. Complete cytoreduction, however, remains crucial, as it provides the best foundation for achieving optimal long-term outcomes and maximizing the benefits of maintenance therapy.

## 1. Introduction

Ovarian cancer remains a significant global health challenge, ranking as the eighth most common cancer among women and the leading cause of death from gynecologic malignancies. According to the most recent GLOBOCAN 2022 estimates, approximately 324,600 new ovarian cancer cases were diagnosed worldwide. In the United States alone, an estimated 20,890 new cases and 12,730 deaths are expected to occur in 2025 [1,2]. The vast majority of these malignancies are of epithelial origin, comprising various histological subtypes such as high-grade serous, endometroid, and clear cell carcinoma [3]. A critical challenge in managing epithelial ovarian cancer (EOC) is the lack of effective early detection methods [3]. Consequently, approximately 75% of patients are diagnosed at an advanced stage (FIGO Stage III or IV), where cancer has already disseminated throughout the peritoneal cavity, significantly complicating treatment and worsening prognosis [4,5].

Standard management for advanced EOC is cytoreductive surgery combined with platinum-based chemotherapy [3]. The primary goal is to achieve complete cytoreduction, defined as the absence of any macroscopic residual disease. Despite the goal of achieving a complete cytoreduction, the unique way ovarian cancer spreads throughout the abdomen, the location of tumors, and the balance between surgical radicality and acceptable morbidity create situations where leaving some residual disease behind is unavoidable [6,7]. Therefore, despite primary treatment combining surgery and chemotherapy, recurrence rates remain high with up to 70% of patients with advanced disease experiencing a relapse [8], which underscore the urgent need for more effective long-term strategies.

Poly (ADP-ribose) polymerase (PARP) inhibitors have been a cornerstone in the treatment for EOC, particularly as a maintenance therapy following platinum-based chemotherapy [7]. By targeting deficiencies in the homologous recombination repair pathway, most notably in tumors with BRCA1/2 mutations [9], PARP inhibitors have demonstrated a significant improvement in progression-free survival (PFS) across multiple clinical trials [10,11,12,13,14,15,16].

While the efficacy of PARP inhibitors is well-established, a critical question is whether their benefit is uniform across all patient subgroups. The use of PARP inhibitors in this setting—where patients present with varying levels of postoperative residual disease—necessitates a rigorous evaluation of whether their therapeutic benefit is maintained or is, in fact, modulated by the initial surgical outcome. Recent real-world evidence reinforces the prognostic importance of complete cytoreduction [17]; however, its role as a predictive factor for PARP inhibitor efficacy remains uncertain. Although most pivotal first-line trials have reported subgroup analyses by surgical outcome, these studies were not specifically designed or sufficiently powered to determine whether the extent of cytoreduction modifies treatment efficacy. Therefore, a quantitative synthesis is warranted to clarify whether residual disease modifies the efficacy of PARP inhibitor maintenance therapy. Understanding this interaction is clinically important, as it could optimize patient selection for those most likely to benefit from PARP inhibitor maintenance therapy and guide clinical decision-making for a large proportion of women with advanced EOC.

PFS was selected as the primary endpoint because it represents the most direct measure of PARP inhibitor activity in the first-line maintenance setting. All major phase III trials of PARP inhibitors in advanced ovarian cancer [10,11,12,13,14,15,16] designated PFS as the primary outcome, enabling reliable comparison across studies. In contrast, overall survival was not fully reported in all trials and is affected by subsequent therapies received after disease progression, which limit its ability to isolate the treatment effect of maintenance therapy [18]. Therefore, evaluating PFS provides a more accurate assessment of whether residual disease status modifies the efficacy of PARP inhibitors in this population.

This systematic review and meta analysis of randomized controlled trials (RCTs) aimed to determine the impact of residual disease status on the efficacy of PARP inhibitors in patients with advanced epithelial ovarian cancer. Specifically, we assessed PFS outcomes according to residual disease status, with additional stratification by clinical risk and timing of surgery.

## 2. Materials and Methods

### 2.1. Search Strategy and Selection Criteria

This systematic review and meta analysis analyzed RCTs that evaluated the PARP inhibitor efficacy, as measured by PFS, stratified by residual disease subgroup. This review was performed in accordance with the PRISMA (Preferred Reporting Items for Systematic Reviews and Meta-Analyses) guidelines and has not been registered [19]. We conducted a systematic search of PubMed, EMBASE, and the Cochrane Library for full-text articles and abstracts published up to July 2, 2025. The following search terms were used: (“Ovarian Neoplasms” OR “Carcinoma, Ovarian Epithelial”) AND (“Poly(ADP-ribose) Polymerase Inhibitors” OR “olaparib” OR “niraparib” OR “rucaparib”) AND “Maintenance therapy” AND (“Neoplasm, Residual” OR “Cytoreduction Surgical Procedures”) AND “Progression-Free Survival”. The search strategy for each database is detailed in Table S1.

**Figure 1:**
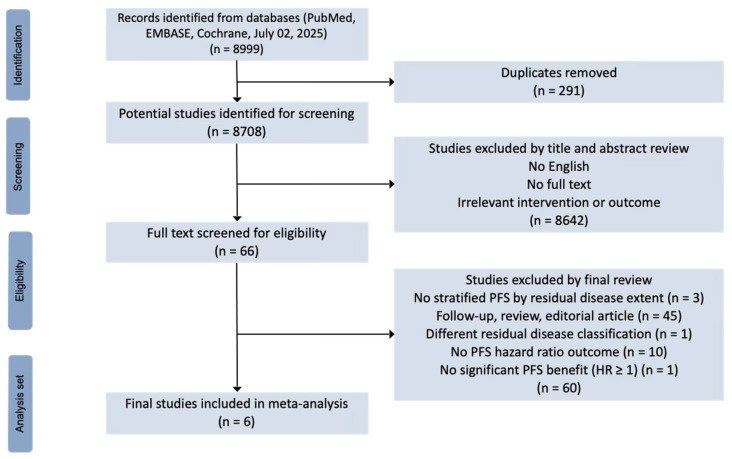
Preferred Reporting Items for Systematic Reviews and Meta-Analyses (PRISMA) flow diagram.

Studies were screened and selected according to predefined eligibility criteria structured around the Patient, Intervention, Comparison, and Outcome (PICO) framework. Patients included in the study were those with newly diagnosed FIGO stage III or IV epithelial ovarian, fallopian tube, or primary peritoneal cancer, and those who achieved a complete or partial response after first-line platinum-based chemotherapy. Intervention was PARPi maintenance therapy while the comparator was placebo. The primary outcome was the hazard ratio for progression-free survival, comparing patients in subgroups stratified by residual disease status, clinical risk, and timing of surgery. Studies were excluded if they met any of the following criteria: (1) studies without full-text availability, (2) studies lacking progression-free survival HRs stratified by residual disease subgroups, (3) studies using different residual disease classification (Figure 1). Only full-text, peer-reviewed articles published in English were included. Conference abstracts, unpublished data, and gray literature were not considered because they lacked sufficient information for subgroup-specific hazard ratio extraction.

### 2.2. Data Analysis

Two independent reviewers screened all studies and extracted data. Any discrepancies were first resolved through discussion, and if consensus was not reached, a senior author served as an independent adjudicator to make the final decision. Data were extracted using a standardized form developed a priori to ensure consistency across reviewers, including study characteristics, interventions, and subgroup-specific hazard ratios. This meta-analysis was conducted at the trial level using published aggregate data rather than individual patient-level data, as patient-level datasets from the included randomized trials were not publicly available. Subgroup analyses were performed based on reported HRs from each study. The following data were extracted from each study: author names, year of publication, type of intervention, number of enrolled patients, HRs for progression-free survival and overall survival with 95% confidence intervals.

The primary endpoint was the pooled HRs for progression-free survival. Subgroup analyses were performed to evaluate treatment efficacy based on residual disease status (no gross residual vs. residual). No gross residual disease was defined as R0, while macroscopic residual disease group included both microscopic (R1) and macroscopic (R2) residual disease. Additional subgroup analysis was conducted to compare PFS outcomes by clinical risk (higher risk vs. lower risk). Higher-risk patients were defined as those with stage IV disease or those with stage III disease who had either residual disease following primary cytoreductive surgery or had undergone interval surgery. Lower-risk patients were defined as those with stage III disease who did not have residual disease following primary cytoreductive surgery [14,20,21]. Further comparison was made between patients who underwent primary cytoreductive surgery (PCS) and those who underwent interval cytoreductive surgery (ICS). These analyses aimed to investigate whether the extent of residual disease contributed to the PFS benefit provided by PARPi maintenance therapy and to identify the prognostic impact of complete cytoreduction.

Univariate HRs were used for meta analysis because multivariable-adjusted HRs were not consistently reported across trials, preventing a comparative analysis. All included studies reported hazard ratios for PFS for both the overall study population and predefined subgroups, and both were included in the meta analysis. Subgroup-specific HRs from the same trial were treated as independent data points in the pooled analyses, consistent with previous meta-analytic approaches. However, because subgroup estimates from the same trial are not fully independent, we analyzed them separately across trials and did not combine within-trial subgroups in the same model to minimize potential dependency. This approach was necessary because covariance data between subgroups were not available from the published trials, precluding the use of multivariate models. Heterogeneity among studies was assessed using Cochran’s Q statistic and Higgins’ I^2^ statistic [22]. A Higgins’ I^2^ value greater than 50% was considered indicative of substantial heterogeneity and in such cases, pooled HRs were calculated using a random-effects model according to the DerSimonian and Laird method [23].

In the VELIA trial, HRs for PFS were reported for subgroups that combined residual disease status with the timing of surgery [12]. To facilitate a subgroup analysis based solely on residual disease status without bias, we pooled the data from the relevant subgroups within the study. The combined HR and its 95% confidence interval were calculated using the inverse-variance weighted average method [24], as recommended by the Cochrane Handbook for Systematic Reviews of Interventions (Chapter 10.3.3) [25]. A combined log HR was then generated using the following formula, where the weight ($\omega_{i}$) is the inverse of the variance ($\frac{1}{{SE}_{i}^{2}}$) of each estimate: ${logHR}_{combined}=\frac{\sum \omega_{i}\cdot {logHR}_{i}}{\sum \omega_{i}},\omega_{i}=\frac{1}{{SE}_{i}^{2}}$. The resulting pooled log HR and its standard error were then transformed back to the natural scale to obtain a single combined HR and 95% confidence interval for each residual disease category, which was used in our meta analysis.

To explore whether the residual disease status, clinical risk, or timing of surgery modified the effect of PARPi maintenance therapy, the significance of the difference between the subgroups was assessed using the chi-squared (Q) test for heterogeneity. A *p*-value of less than 0.05 for the Q-test was considered indicative of a statistically significant subgroup difference [26]. Meta-regression was not feasible due to the limited number of included studies (*n* = 6), as it would have been underpowered and could have yielded unreliable results [27]. To assess the robustness of the pooled estimates, sensitivity analyses were performed, including a leave-one-out analysis excluding each study in turn and an alternative analysis incorporating harmonized residual disease definitions.

Publication bias was evaluated using funnel plots, where the effect size was plotted on the x-axis, and the standard error was plotted on the y-axis. Study quality was assessed using the Risk of Bias-2 (RoB-2) tool, as all included studies were randomized controlled trials (Table S2). The certainty of evidence for each outcome was further evaluated using the GRADE (Grading of Recommendations, Assessment, Development, and Evaluations) approach, which considers risk of bias, inconsistency, indirectness, imprecision, and publication bias [28].

All statistical analyses were conducted using R software version 4.5.1 and a two-sided *p*-value < 0.05 was considered statistically significant. In accordance with the journal’s guidelines, we will provide our data for independent analysis by a selected team by the Editorial Team for the purposes of additional data analysis or for the reproducibility of this study in other centers if such is requested.

## 3. Results

Six randomized controlled trials (SOLO1, PAOLA-1, VELIA, ATHENA-MONO, PRIMA, FLAMES), encompassing a total of 3629 patients and published up to 2 July 2025, were identified and included in this meta analysis (Figure 1). Olaparib (monotherapy in SOLO1 and with bevacizumab in PAOLA-1) was tested in two of these studies, while veliparib, rucaparib, niraparib, and senaparib were tested in the remaining trials. All trials demonstrated a low risk of bias across all domains of the Cochrane RoB-2 tool: randomization process, deviations from intended interventions, missing outcome data, measurement of the outcome, and selection of the reported result (Table S2). To explore the potential for publication bias, funnel plots were generated for each subgroup (Figure S1). The plot for the R0 group appeared reasonably symmetrical, while some asymmetry was observed in the R1/R2 group. However, it is critical to acknowledge that with a small number of included studies (k = 6), visual interpretation of a funnel plot must be made with caution.

To assess the robustness of our findings, a leave-one-out sensitivity analysis was conducted. This analysis confirmed that the pooled HRs for progression-free survival were highly stable and not unduly influenced by any single study. The overall conclusion remained consistent even after excluding the SOLO1 trial, which exclusively enrolled patients with BRCA mutations and had the most substantial treatment effect. This stability strengthens the confidence in our finding that PARP inhibitors provide a significant clinical benefit across the broader patient groups included in this meta analysis (Figure S2). Furthermore, a separate sensitivity analysis was performed to include the PRIME trial, which used a different classification for residual disease. For this analysis, we created harmonized subgroups: ‘No/Microscopic Residual Disease’ (defined as R0 for all trials except PRIME, where it was R0/R1) and ‘Visible/Macroscopic Residual Disease’ (defined as R1/R2 for all trials except PRIME, where it was R2). The pooled HRs were very similar between these two harmonized groups (No/microscopic = 0.53, 95% CI: 0.42–0.68; Visible/macroscopic = 0.49, 95% CI: 0.35–0.68). This indicates that our primary finding—that the PARP inhibitors are effective regardless of residual disease status—remained robust in a sensitivity analysis that included the PRIME trial using these harmonized subgroup definitions (Figure S3).

GRADE assessment confirms that while there is moderate certainty in the overall benefit of PARP inhibitors for patients with both R0 and R1/R2 disease, the certainty of evidence for a difference in efficacy between these two groups is low. This low certainty is driven by moderate-to-substantial heterogeneity across studies (I^2^ > 50%) and a lack of statistical precision (Table 1).

The characteristics of the included studies, including study design, intervention details, patient population, and key outcome measures, are summarized in Table 2. Our meta analysis confirmed that PARPi maintenance therapy provides a significant PFS benefit in patients with newly diagnosed advanced ovarian cancer. To investigate the consistency of this benefit across different patient populations, several pre-specified subgroup analyses were conducted.

**Figure 2:**
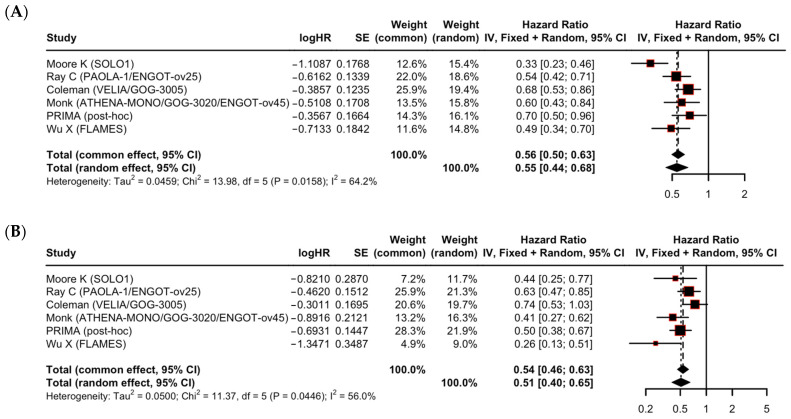
Subgroup analysis of progression-free survival by residual disease status (**A**) R0 and (**B**) R1/R2 [10,11,12,13,14,15,16]. Each square represents the hazard ratio (HR) estimate for an individual study, with the size of the square proportional to the study’s weight in the meta-analysis. The diamond represents the pooled HR, with its width indicating the 95% CI for the overall effect. No significant difference was observed between subgroups (*p* = 0.66).

A subgroup analysis demonstrated that PARPi maintenance therapy significantly improved PFS regardless of residual disease status after cytoreductive surgery. In the subgroup of patients with no residual disease (R0), the pooled HR was 0.55 (95% CI: 0.44–0.68). In patients with macroscopic residual disease (R1/R2), the pooled HR was 0.51 (95% CI: 0.40–0.65) (Figure 2). The difference in the treatment effect between these two subgroups was not statistically significant (*p* = 0.66 for interaction) (Table 3). Substantial statistical heterogeneity was noted in both the R0 subgroup (I^2^ = 64.2%) and the R1/R2 subgroup (I^2^ = 56.0%).

**Figure 3:**
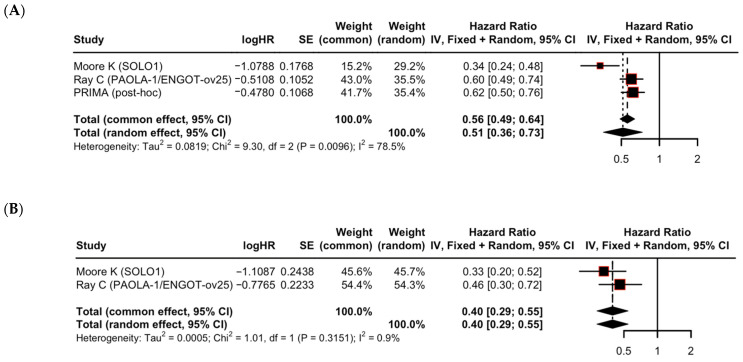
Subgroup analysis of progression-free survival by clinical risk (**A**) higher risk and (**B**) lower risk [10,11,14,15]. Each square represents the hazard ratio (HR) estimate for an individual study, with the size of the square proportional to the study’s weight in the meta-analysis. The diamond represents the pooled HR, with its width indicating the 95% CI for the overall effect.

When stratified by baseline clinical risk, PARPi therapy was numerically more effective in lower-risk populations (HR 0.40, 95% CI: 0.29–0.55) compared to higher-risk populations (HR 0.51, 95% CI: 0.36–0.73). The lack of statistical significance in subgroup comparison (*p* = 0.30) suggests that clinical benefit extends across risk groups (Table 3). A marked difference in heterogeneity was observed between these subgroups. The lower-risk group showed high consistency across trials (I^2^ = 0.9%), while the higher-risk group exhibited substantial heterogeneity (I^2^ = 78.5%) (Figure 3).

The PFS benefit of PARPi maintenance therapy was consistent for patients regardless of the timing of their surgery. The pooled HR was 0.56 (95% CI: 0.42–0.74) for patients who underwent primary cytoreductive surgery and 0.54 (95% CI: 0.45–0.66) for those who received interval cytoreductive surgery. There was no significant difference in treatment effect between the two groups. High heterogeneity was observed in the PCS subgroup (I^2^ = 72.3%), and moderate heterogeneity was noted in the ICS subgroup (I^2^ = 44.2%) (Figure S4).

Individual trial data confirm that patients with no residual disease (R0) consistently showed superior outcomes compared to those with residual disease (R1/R2). In the PRIMA trial, median PFS with niraparib was 18.2 months for R0 versus 11.2 months for R1/R2 (Table S3). Similar patterns were observed in placebo arms across trials, confirming residual disease as an independent prognostic factor.

## 4. Discussion

### 4.1. Summary of Main Results

This systematic review and meta analysis comprising six trials enrolling a total of 3629 patients demonstrated that PARP inhibitor maintenance therapy provides a robust and statistically significant PFS benefit for patients with newly diagnosed advanced ovarian cancer across key clinical and surgical subgroups. We found no significant difference in the relative treatment effect based on a patient’s residual disease status (R0 vs. R1/R2), or the timing of their cytoreductive surgery (primary vs. interval). However, the absence of statistical significance does not equate to the absence of a true difference; rather, it reflects the limited statistical power of subgroup analyses based on trial-level data. While the PFS benefit was slightly more significant in lower- compared to higher-risk clinical group, there was a notable difference in the consistency of the effect. The treatment benefit was highly uniform among lower-risk patients, whereas it was highly variable among higher-risk patients.

### 4.2. Results in the Context of Published Literature

This meta analysis provides robust, pooled confirmation that PARP inhibitor maintenance therapy delivers significant PFS benefit across key clinical subgroups, as reported in individual trials [10,11,12,13,14,15,16]. This meta analysis gives this conclusion greater statistical power, solidifying that the significant relative PFS benefit is maintained regardless of the patient’s residual disease status, clinical risk, or the timing of their cytoreductive surgery.

This consistency leads to a crucial distinction that forms the central thesis of our discussion: the distinction between factors that determine a patient’s absolute prognosis and those that predict the relative benefit of PARP inhibitor therapy. Our analysis demonstrates that while clinical and surgical factors are powerful prognostic indicators, they do not appear to be strong predictive markers for the efficacy of PARP inhibitors.

This distinction is best illustrated by our analysis of heterogeneity. The striking difference between clinical risk subgroups is a critical finding (Figure 3). Low heterogeneity in the lower-risk group (I^2^ = 0.9%) suggests a uniform and predictable benefit in a favorable clinical population. Conversely, the substantial heterogeneity in the higher-risk group (I^2^ = 78.5%) suggests that while higher-risk patients benefit, the magnitude of that benefit is likely governed not by clinical risk alone, but also by underlying biological drivers. This is exemplified by the large effect size of the SOLO1 trial, which exclusively enrolled patients with BRCA mutations and was a major contributor to this heterogeneity. The inclusion of different PARP inhibitors (olaparib, niraparib, and rucaparib), with distinct pharmacological properties, may also contribute to the observed variability in the pooled treatment effect.

A review of the mPFS from key trials (SOLO1, PAOLA-1, and PRIMA) underscores the prognostic power of these clinical factors. Patients with no residual disease (R0) and lower-risk consistently demonstrate a substantially longer mPFS in both the placebo and PARPi arms than their R1/R2 and higher-risk counterparts [10,11,14,15,20,29]. This confirms that achieving an R0 status is a foundational goal for improving a patient’s overall prognosis. The clinical implication is profound; even patients with a poorer prognosis, such as those with macroscopic residual disease or higher clinical risk, derive a similar relative benefit from PARPi and should therefore be strongly considered for this therapy.

Ultimately, these findings lead to two complementary and equally critical messages for clinicians. First, the pursuit of complete cytoreduction to R0 remains a primary surgical goal to optimize a patient’s baseline prognosis. Second, PARP inhibitor maintenance therapy is a powerful therapeutic strategy that provides a significant relative benefit to all eligible patients, building upon a favorable surgical outcome or, perhaps more critically, providing a crucial risk reduction even when a complete resection is not achieved. Interestingly, an exploratory analysis of the individual hazard ratios suggesting a slight numerical trend towards a greater relative benefit in the R1/R2 subgroup reinforces this second point. Specifically, using an original VELIA trial data disaggregated by surgical timing (PCS vs. ICS), we noted that four of the seven study comparisons numerically confirmed greater benefit in the R1/R2 subgroup, while three favored the R0 subgroup. This observation, though requiring cautious interpretation due to the use of disaggregated data, highlights that patients with the poor prognosis may have the most to gain in relative terms from an effective therapy. Therefore, to optimize patient outcomes in advanced ovarian cancer, both goals—achieving R0 status and offering PARPi maintenance regardless of surgical outcome—must be pursued.

### 4.3. Strengths and Weaknesses

This meta-analysis possesses several key strengths. It is, to our knowledge, the most up-to-date meta-analysis that incorporates all pivotal, contemporary Phase III trials that discuss PARP inhibitor maintenance therapy. This ensures our findings are based on a high level of evidence relevant to current clinical practice. Methodological rigor was maintained by employing the Cochrane RoB2 tool and confirming the stability of our conclusions through multiple sensitivity analyses. The use of GRADE framework provides a transparent assessment of the certainty of our evidence. A key conceptual strength of our work is the detailed analysis distinguishing between a factor’s prognostic value and its ability to predict relative treatment benefit, which helps clarify the complementary roles of surgery and maintenance therapy.

At the same time, we acknowledge several limitations. First, our analysis was limited to aggregate data reported by the individual trials. The lack of individual patient data (IPD) prevented more granular analyses, such as exploring the interplay between multiple factors simultaneously (e.g., the combined effect of HRD status and residual disease) and bound our work to the subgroup definitions used in the original trials. Second, significant statistical heterogeneity was observed in several analyses. This is likely multifactorial, stemming from key differences between the trials, including different PARP inhibitors, different treatment regimens (e.g., the inclusion of bevacizumab as background therapy in PAOLA-1) and different patient populations, particularly regarding biomarker status. Consequently, the pooled estimates represent an average effect across diverse settings, and caution must be used when applying them to a specific patient. Finally, subgroup-specific hazard ratios from the same trial were treated as independent data points because patient-level data were unavailable. This approach, commonly used in aggregate-level meta-analyses, may have introduced minor dependency between subgroups, although sensitivity analyses confirmed that the main conclusions were not affected. The lower-risk subgroup analysis was limited to two trials (SOLO1 and PAOLA-1), as this specific risk stratification was not uniformly reported across all studies. Although these trials represent a large number of patients and showed a highly consistent effect, the small number of studies precludes a definitive conclusion about the generalizability of this finding. The robustness of this result would be significantly strengthened as more data becomes available.

### 4.4. Implications for Practice and Future Research

Our findings have significant implications for the clinical management of patients with advanced epithelial ovarian cancer. This meta analysis provides robust evidence that the relative benefit of PARP inhibitor maintenance therapy is not dependent on the outcome of primary cytoreductive surgery. The primary clinical message is that residual disease status, while a powerful prognostic factor for survival, should not be used as a predictive biomarker to guide the use of PARP inhibitors, thereby answering a critical question of clinical uncertainty.

While our findings demonstrate that PARP inhibitor efficacy is maintained regardless of residual disease status, this should not alter the primary surgical goal of achieving complete cytoreduction. Clinicians must counsel patients that despite the robust efficacy of PARP inhibitors across surgical outcomes, maximal cytoreductive effort remains essential for optimal baseline prognosis. The availability of effective maintenance therapy does not diminish the importance of pursuing R0 resection, particularly in patients with BRCA mutations or HRD who are most likely to benefit from PARP inhibitors.

Our findings clarify residual disease as a prognostic rather than predictive factor for PARP inhibitor therapy, emphasizing the need for better biomarkers beyond BRCA status. Long-term follow-up will determine if PFS benefits translate to overall survival gains, particularly in patients with residual disease.

Future clinical trials should be prospectively designed to report outcomes stratified by both residual disease and molecular biomarkers. Such an approach will be essential to truly personalize treatment and optimize the integration of PARP inhibitors into the ovarian cancer therapeutics landscape. Ultimately, a future individual patient data (IPD) meta analysis is warranted to definitively address the heterogeneity observed across trials. An IPD meta analysis would enable multivariable analysis to untangle the combined effects of residual disease status and HRD status, as well as the interplay between PARP inhibitor type and other patient-level factors, providing the highest level of evidence to truly personalize therapy.

## 5. Conclusions

PARP inhibitor maintenance therapy significantly improves progression-free survival in advanced epithelial ovarian cancer. The benefit was consistent across subgroups stratified by residual disease status, suggesting that residual disease does not markedly modify the efficacy of PARP inhibitors. These findings underscore that residual disease should not preclude eligible patients from receiving PARP inhibitor maintenance, as substantial therapeutic benefit is consistently maintained across surgical outcomes. Importantly, while the magnitude of benefit does not appear to differ by residual disease status, achieving complete cytoreduction remains a cornerstone of optimal management because it confers the most favorable baseline prognosis and maximizes the absolute gains from maintenance therapy.

## Figures and Tables

**Table 1:** GRADE Summary of Findings. Symbols: ⊕ indicates a point of confidence in the evidence, while ⊖ indicates a reduction in confidence according to the GRADE framework. N/A = Not applicable.

Outcomes	No. of Studies (Participants)	Pooled Effect (95% CI)	Starting Certainty	Downgrading Factors	Certainty of the Evidence (GRADE)
PFS benefit in patients with no residual disease (R0)	6 RCTs (*n* = 2015)	HR 0.55 (95% CI 0.44–0.68)	High	Downgraded one level due to concerns regarding inconsistency (I^2^ = 64.2%).	Moderate ⊕⊕⊕⊖
PFS benefit in patients with macroscopic residual disease (R1/R2)	6 RCTs (*n* = 1093)	HR 0.51 (95% CI 0.40–0.65)	High	Downgraded one level due to concerns regarding inconsistency (I^2^ = 56.0%).	Moderate ⊕⊕⊕⊖
Difference in effect between R0 and R1/R2 subgroups	N/A	Test for difference: *p* = 0.66	High	Downgraded two levels due to concerns regarding inconsistency and concerns regarding imprecision (insufficient power to rule out a clinically important difference)	Low ⊕⊕⊖⊖

**Table 2:** Descriptions of trials included in the meta analysis. (A) Characteristics of included trials and overall progression-free survival (PFS) and overall survival (OS) outcomes. (B) Subgroup hazard ratios for progression-free survival by residual disease status, clinical risk, and timing of surgery.

**(A)**							
**Trial**	**Study Design**	**N (Experimental Arm)**	**N (Control Arm)**	**Primary Endpoint**	**Secondary Endpoint**	**HR for PFS (95% CI) Whole Population**	**OS HR (95% CI)**
Moore K (SOLO1), 2018 [10]	Phase III RCT, double-blind, placebo-controlled	260, Olaparib	131, Placebo	PFS	OS, PFS2, time until first and second subsequent therapies, QoL	0.30 (0.23–0.41)	0.55 (0.40–0.76)
Ray C (PAOLA-1/ENGOT-ov25), 2019 [11]	Phase III randomized, double-blind, placebo-controlled	537, Olaparib + Bev *	269, Placebo + Bev *	PFS	OS, PFS2, Time until subsequent therapy, QoL	0.59 (0.49–0.72)	-
Coleman (VELIA/GOG-3005), 2019 [12]	Phase III, randomized, double-blind, placebo-controlled trial	382, Veliparib throughout	375, Placebo	PFS	OS, NFOSI-18 symptom score	0.68 (0.56–0.83)	-
Monk (ATHENA-MONO/GOG-3020)/ENGOT-ov45), 2022 [13]	Phase III, randomized, double-blind	427, Rucaparib	111, Placebo	PFS	OS, ORR, Duration of response, Safety	0.52 (0.40–0.68)	-
O’Cearbhaill (PRIMA post-hoc), 2019 [14,15]	Phase III, double-blind, placebo-controlled	487, Niraparib	246, Placebo	PFS	-	0.62 (0.50–0.76)	1.01 (0.84–1.23)
Wu X (FLAMES), 2024 [16]	Phase III, randomized, double-blind	271, Senaparib	133, Placebo	PFS	OS, PFS2, Time until subsequent therapy/death, QoL	0.43 (0.31–0.58)	-
**(B)**							
**Trial**	**HR for PFS (95% CI)** **Higher Risk**	**HR for PFS (95% CI)** **Lower Risk**	**HR for PFS (95% CI)** **R0**		**HR for PFS (95% CI) R** **1/R2**	**HR for PFS (95% CI)** **PCS**	**HR for PFS (95% CI)** **ICS**
Moore K (SOLO1), 2018 [10]	0.34 (0.24–0.48)	0.33 (0.20–0.52)	0.33 (0.23–0.46)		0.44 (0.25–0.77)	0.31 (0.21–0.46)	0.37 (0.24–0.58)
Ray C (PAOLA-1/ENGOT-ov25), 2019 [11]	0.60 (0.49–0.74)	0.46 (0.30–0.72)	0.54 (0.42–0.71)		0.63 (0.47–0.85)	0.52 (0.40–0.69)	0.66 (0.50–0.87)
Coleman (VELIA/GOG-3005), 2019 [12]	-	-	After PDS: 0.77 (0.58–1.04)/ After IDS: 0.52 (0.34–0.81)		After PDS: 0.60 (0.40–0.91)/ After IDS: 1.12 (0.62–2.00)	0.72 (0.57–0.92)	0.64 (0.45–0.90)
Monk (ATHENA-MONO/GOG-3020)/ENGOT-ov45), 2022 [13]	-	-	0.60 (0.43–0.84)		0.41 (0.27–0.62)	0.64 (0.43–0.95)	0.44 (0.31–0.62)
O’Cearbhaill (PRIMA post-hoc), 2019 [14,15]	0.62 (0.50–0.76)	-	0.70 (0.50–0.96)		0.50 (0.38–0.67)	0.67 (0.47–0.96)	0.57 (0.44–0.73)
Wu X (FLAMES), 2024 [16]	-	-	0.49 (0.34–0.70)		0.26 (0.13–0.51)	-	-

* Bev = Bevacizumab.

**Table 3:** Summary of subgroup analyses and test for differences.

Subgroup Analysis	Subgroups Compared	Number of Studies (k)	Pooled HR (95% CI) per Subgroup	Heterogeneity (I^2^)	*p*-Value for Subgroup Difference
Residual disease	R0	6	0.55 (0.44–0.68)	64.2%	0.66
	R1/R2	6	0.51 (0.40–0.65)	56.0%	
Clinical risk	Higher risk	3	0.51 (0.36–0.73)	78.5%	0.30
	Lower risk	2	0.40 (0.29–0.55)	0.9%	
Timing of surgery	PCS	5	0.56 (0.42–0.74)	72.3%	0.88
	ICS	5	0.54 (0.45–0.66)	44.2%	

## Data Availability

Data are available upon request.

## Supplementary Materials

The following supporting information can be downloaded at https://www.mdpi.com/article/10.3390/cancers17203365/s1, Figure S1: Funnel plot for evaluating publication bias stratified by residual disease (A) R0 (B) R1/R2; Figure S2: Sensitivity analysis by Leave-One-Out method; Figure S3: Sensitivity analysis of progression-free survival by harmonized residual disease status, including the PRIME trial; Figure S4: Subgroup analysis of progression-free survival by timing of surgery (A) PCS (B) ICS; Table S1: Search strategy; Table S2: Quality assessment using the Cochrane collaboration tool (ROB-2) for six randomized controlled trials; Table S3: Median progression-free survival (mPFS) by prognostic subgroups in key trials [15,20,29].
