# Supplementary material for: Influence of Residual Disease on the Efficacy of PARP Inhibitors in Advanced Epithelial Ovarian Cancer: A Systematic Review and Meta Analysis
Source: Cancers (Basel). 2025 Oct 18;17(20):3365. doi: 10.3390/cancers17203365 (PMC12562901; doi:10.3390/cancers17203365)
Supplement: Supplementary file 1 [file cancers-17-03365-s001.zip › cancers-3916831-supplementary.pdf]

**Table S1.** Search strategy.

**Search strategy for Pubmed**

#1. “Ovarian Neoplasms” [MeSH Terms]

#2. “Carcinoma, Ovarian Epithelial” [MeSH Terms]

#3. (#1) OR #2

#4. "Poly(ADP-ribose) Polymerase Inhibitors" [MeSH Terms]

#5. “olaparib”

#6. “niraparib”

#7. “rucaparib”

#8. (((#4) OR #5) OR #6) OR #7

#9. “Maintenance therapy”

#10. “Neoplasm, Residual” [MeSH Terms]

#11. "Cytoreduction Surgical Procedures" [MeSH Terms]

#12. (#10) OR #11

#13. “Progression-Free Survival” [MeSH Terms]

#14. ((((#3) AND #8) AND #9) AND #12) AND #13

Date of Search: Jul 2, 2025

Result: 164 articles found

**Search strategy for EMBASE**

#1. 'ovary carcinoma'/exp

#2. 'ovary cancer'/exp

#3. #1 OR #2

#4. 'nicotinamide adenine dinucleotide adenosine diphosphate ribosyltransferase inhibitor'/exp
#5. ‘olaparib’/exp

#6. ‘niraparib’/exp

#7. ‘rucaparib’/exp

#8. #4 OR #5 OR #6 OR #7

#9. ‘maintenance therapy’/exp

#10. ‘cytoreductive surgery’/exp

#11. ‘residual disease’

#12. #10 OR #11

#13. ‘progression free survival’/exp

#14. #3 AND #8 AND #9 AND #12 AND #13

Date of Search: Jul 2, 2025

Result: 6,821 articles found

**Search strategy for the Cochrane Library**

#1. ‘epithelial ovarian cancer’

#2. ‘PARP inhibitor’

#3. ‘olaparib’

#4. “niraparib’

#5. “rucaparib’

#6. #2 OR #3 OR #4 OR #5

#7. ‘maintenance chemotherapy’

#8. ‘residual disease’

#9. ‘cytoreduction’

#10 #8 OR #9

#11 ‘progression-free survival’

#12 ((((#1) AND #6) AND #7) AND #10) AND #11

Date of Search: Jul 2, 2025

Result: 2014 articles found

**Table S2.** Quality assessment using the Cochrane collaboration tool (ROB-2) for six randomized controlled trials.

| | D1 | D2 | D3 | D4 | D5 | Overall |
|---|---|---|---|---|---|---|
| Moore K (SOLO1), 2018 | + | + | + | + | + | + |
| Ray C (PAOLA-1/ENGOT-ov25), 2019 | + | + | + | + | + | + |
| Coleman (VELIA/GOG-3005), 2019 | + | + | + | + | + | + |
| Monk (ATHENA-MONO/GOG-3020/ENGOT-ov45), 2022 | + | + | + | + | + | + |
| O'Cearbhaill (PRIMA), 2019 | + | + | + | + | + | + |
| Wu X (FLAMES), 2024 | + | + | + | + | + | + |

Domains:

D1 = Randomization process

D2 = Deviations from the intended interventions

D3 = Missing outcome data

D4 = Measurement of the outcome

D5 = Selection of the reported result

(A)

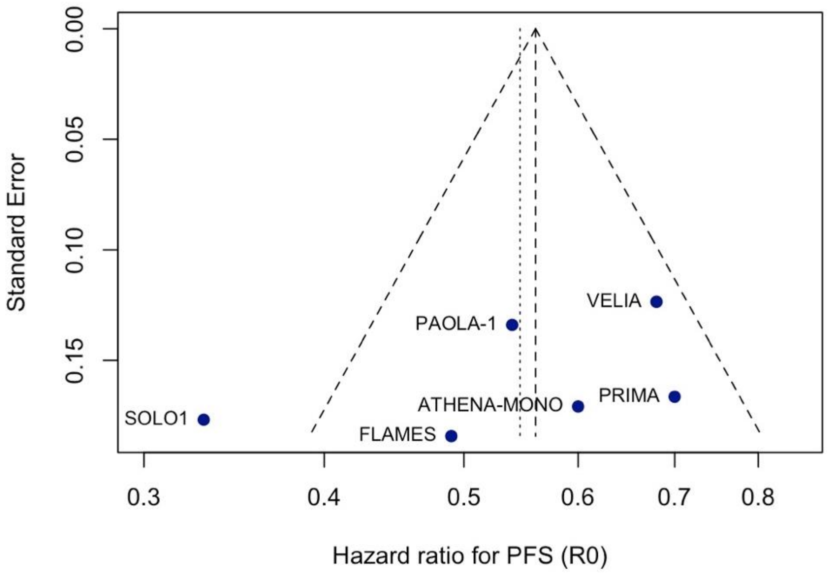


(B)

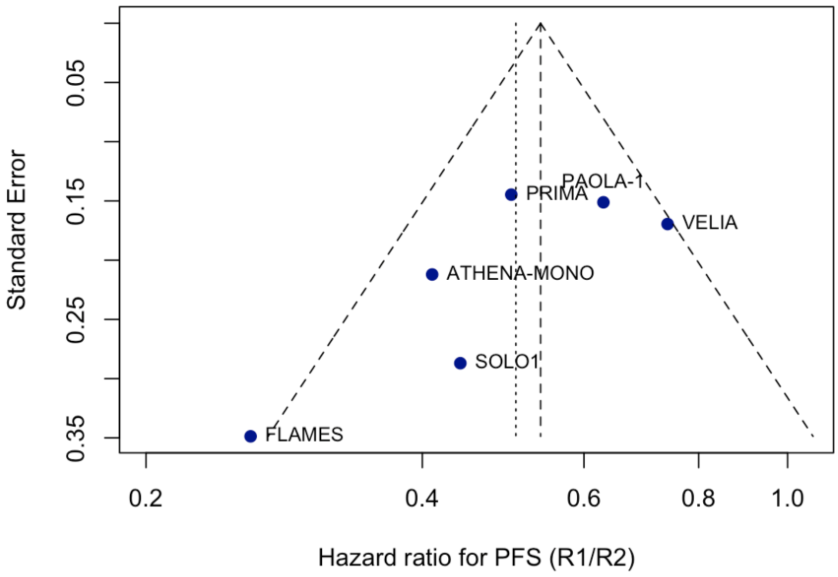


**Figure S1.** Funnel plot for evaluating publication bias stratified by residual disease (A) R0 (B) R1/R2.

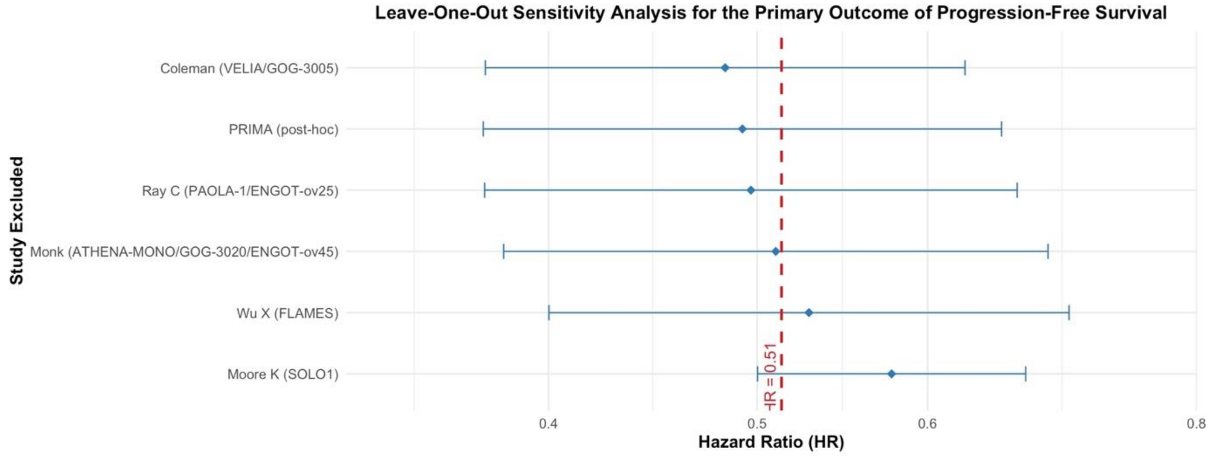


**Figure S2.** Sensitivity analysis by Leave-One-Out method.

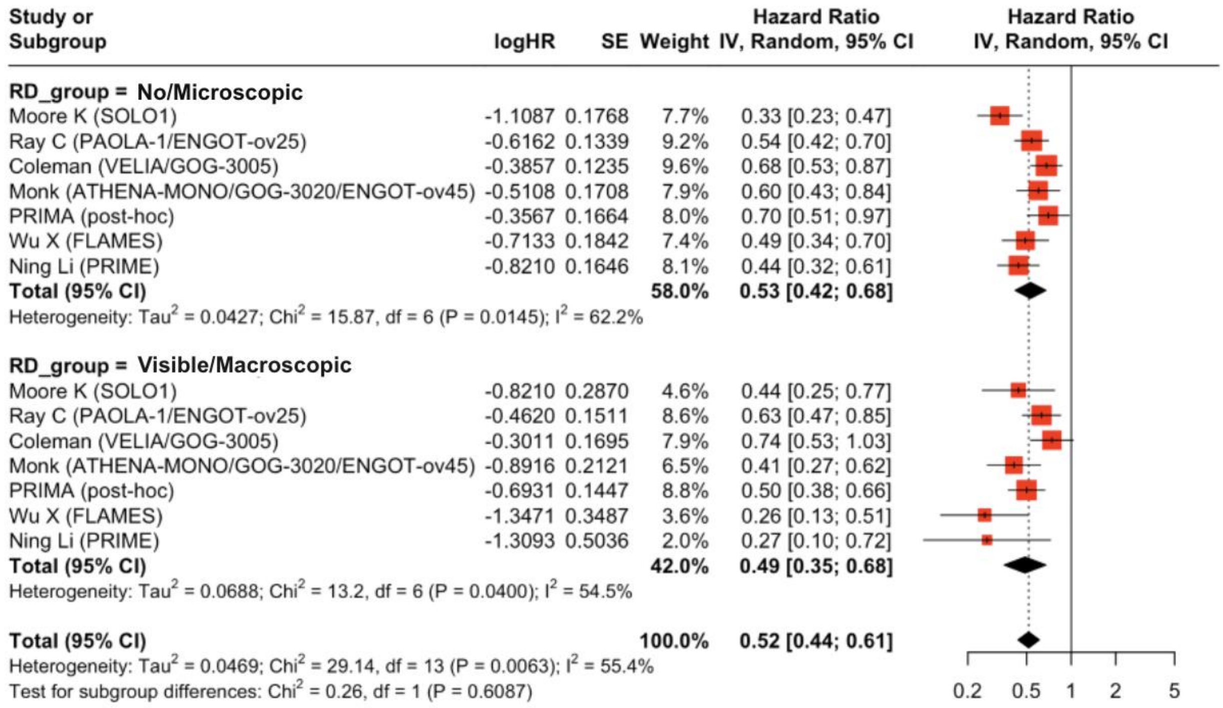


**Figure S3.** Sensitivity analysis of progression-free survival by harmonized residual disease status, including the PRIME trial.

(A)

| Study | logHR | SE | Weight (common) | Weight (random) | Hazard Ratio IV, Fixed + Random, 95% CI |
|---|---|---|---|---|---|
| Moore K (SOLO1) | -1.1712 | 0.2000 | 12.6% | 18.0% | 0.31 [0.21; 0.46] |
| Ray C (PAOLA-1/ENGOT-ov25) | -0.6539 | 0.1391 | 26.1% | 21.9% | 0.52 [0.40; 0.69] |
| Coleman (VELIA/GOG-3005) | -0.3285 | 0.1221 | 33.8% | 23.0% | 0.72 [0.57; 0.92] |
| Monk (ATHENA-MONO/GOG-3020/ENGOT-ov45) | -0.4463 | 0.2022 | 12.3% | 17.9% | 0.64 [0.43; 0.95] |
| PRIMA (post-hoc) | -0.4005 | 0.1822 | 15.2% | 19.2% | 0.67 [0.47; 0.96] |
| **Total (common effect, 95% CI)** | | | **100.0%** | . | **0.58 [0.50; 0.67]** |
| **Total (random effect, 95% CI)** | | | . | **100.0%** | **0.56 [0.42; 0.74]** |

Heterogeneity: $Tau^2 = 0.0764$; $Chi^2 = 14.42$, df = 4 (P = 0.0061); $I^2 = 72.3\%$

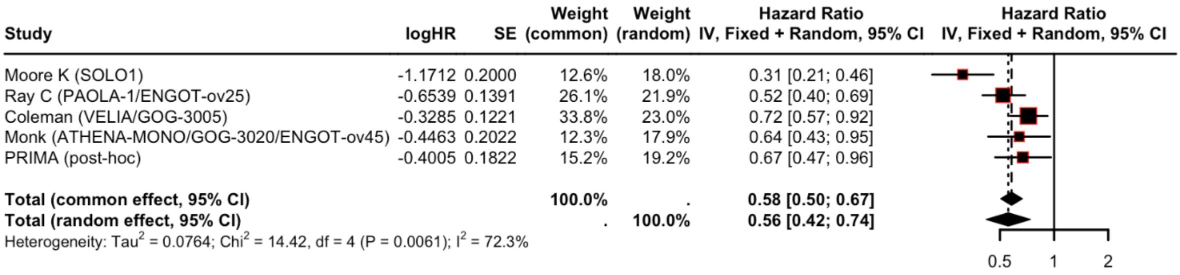


(B)

| Study | logHR | SE | Weight (common) | Weight (random) | Hazard Ratio IV, Fixed + Random, 95% CI |
|---|---|---|---|---|---|
| Moore K (SOLO1) | -0.9943 | 0.2251 | 10.2% | 13.3% | 0.37 [0.24; 0.58] |
| Ray C (PAOLA-1/ENGOT-ov25) | -0.4155 | 0.1413 | 25.9% | 23.8% | 0.66 [0.50; 0.87] |
| Coleman (VELIA/GOG-3005) | -0.4463 | 0.1768 | 16.5% | 18.5% | 0.64 [0.45; 0.90] |
| Monk (ATHENA-MONO/GOG-3020/ENGOT-ov45) | -0.8210 | 0.1768 | 16.5% | 18.5% | 0.44 [0.31; 0.62] |
| PRIMA (post-hoc) | -0.5621 | 0.1292 | 30.9% | 26.0% | 0.57 [0.44; 0.73] |
| **Total (common effect, 95% CI)** | | | **100.0%** | . | **0.55 [0.48; 0.64]** |
| **Total (random effect, 95% CI)** | | | . | **100.0%** | **0.54 [0.45; 0.66]** |

Heterogeneity: $Tau^2 = 0.0192$; $Chi^2 = 7.16$, df = 4 (P = 0.1275); $I^2 = 44.2\%$

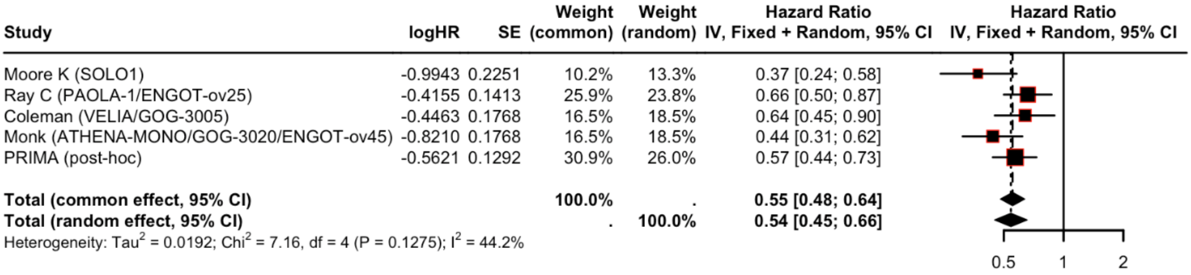


**Figure S4.** Subgroup analysis of progression-free survival by timing of surgery (A) PCS (B) ICS.

**Table S3.** Median progression-free survival (mPFS) by prognostic subgroups in key trials.

| Trial | PARPi | Subgroup | mPFS, months (PARPi) | mPFS, months (Placebo) |
|---|---|---|---|---|
| Moore K (SOLO1), 2018 | Olaparib | R0 (No residual)[1] | NR | 15.3 |
| | | R1/R2 (Residual)[1] | 29.4 | 11.3 |
| | | Higher risk[2] | 40.6 | 11.1 |
| | | Lower risk[2] | NR | 21.9 |
| Ray C (PAOLA-1/ENGOT-ov25), 2019 | Olaparib+Bevacizumab | Higher risk | 20.3 | 14.7 |
| | | Lower risk | 39.3* | 22.9 |
| A. González-Martín (PRIMA), 2019[3] | Niraparib | R0 (No residual) | 18.2 | 11 |
| | | R1/R2 (Residual) | 11.2 | 5.7 |
| | | Higher risk | 13.8 | 8.2 |

NR = Not reached

*Unstable median due to lack of events

[1] DiSilvestro P, Colombo N, Scambia G, Kim BG, Oaknin A, Friedlander M, et al. Efficacy of maintenance olaparib for newly diagnosed, advanced ovarian cancer patients with a BRCA mutation: subgroup analysis findings from the SOLO1 trial. Journal of Clinical Oncology. 2020 Aug 4;

[2] Banerjee S, Moore KN, Colombo N, Scambia G, Kim BG, Oaknin A, et al. Maintenance olaparib for patients with newly diagnosed advanced ovarian cancer and a BRCA mutation (SOLO1/GOG 3004): 5-year follow-up of a randomised, double-blind, placebo-controlled, phase 3 trial. The Lancet Oncology. 2021 Dec;22(12):1721–31.

[3] O'Cearbhaill RE, Jose-Alejandro Pérez-Fidalgo, Monk BJ, Tusquets I, McCormick C, Fuentes J, et al. Efficacy of niraparib by time of surgery and postoperative residual disease status: A post hoc analysis of patients in the PRIMA/ENGOT-OV26/GOG-3012 study. Gynecologic oncology. 2022 Jul 1;166(1):36–43.
